# Periodontitis in a 65-year-old population: risk indicators and impact on oral health-related quality of life

**DOI:** 10.1186/s12903-022-02662-9

**Published:** 2022-12-24

**Authors:** Anne Thea Tveit Sødal, Rasa Skudutyte-Rysstad, My Tien Diep, Odd Carsten Koldsland, Lene Hystad Hove

**Affiliations:** 1 Department of Cariology and Gerodontology, Faculty of Dentistry, University of Oslo, Blindern, P.O. Box 1109, 0317 Oslo, Norway; 2 Department of Periodontology, Faculty of Dentistry, University of Oslo, Oslo, Norway

**Keywords:** Periodontitis, Periodontal diseases, Risk factors, Risk indicators, Oral health-related quality of life, OHIP-14

## Abstract

**Aims:**

The aims of the present study were to investigate socioeconomic and behavioral risk indicators for severe periodontitis in a 65-year-old Norwegian population, and to investigate how periodontitis impacts oral health-related quality of life.

**Material and methods:**

A sample of 65-year-old residents in Oslo, born in 1954, was randomly selected for this study. The participants answered a questionnaire regarding country of birth, education, diabetes, smoking habits, dental attendance pattern, and tooth-brushing habits. In addition, oral health-related quality of life (OHRQoL) was assessed by the Oral Health Impact Profile-14 questionnaire (OHIP-14). Negative impact on OHRQoL was defined as responding “fairly often” or “very often” to at least one of the OHIP-14 items. The diagnosis of periodontitis was based on clinical and radiographic periodontal measurements and classified based on the consensus report from the 2017 World Workshop on the Classification of Periodontal and Peri-Implant Diseases and Conditions.

**Results:**

Of 796 eligible participants, 460 individuals agreed to participate in the present study (response rate 58%). Seven participants were excluded from the analyses due to < 2 remaining teeth (n = 3) or missing questionnaire (n = 4), resulting in a study sample of 453 individuals (233 men and 220 women). An association was found between non-western country of birth, diabetes type 2, lower education, smoking, non-regular dental visits, and severe periodontitis (stage III or IV, n = 163) in bivariate analyses (Chi-square test). However, in the multiple logistic regression model, only non-western country of birth, diabetes type 2 and smoking (former and current), were associated with higher odds of severe periodontitis. The overall mean OHIP-14 total score was 3.6 (SD: 6.1). Participants with stage III or IV periodontitis reported a significantly higher OHIP-14 total score (mean: 4.7, SD: 7.4), indicating a lower OHRQoL, compared to non-periodontitis participants (mean: 2.9, SD: 4.9).

**Conclusions:**

In the present study, non-western birth country, diabetes type 2, and smoking were found as significant risk indicators for severe periodontitis. Overall, results indicate a good OHRQoL among 65-year-olds in Oslo, however, a tendency of reduced OHRQoL with increasing severity of periodontitis was observed.

## Introduction

Periodontitis is an inflammatory disease caused by an immune response due to the presence of pathogenic plaque bacteria in the periodontal tissues [1]. This inflammatory-immune response affects the supporting connective tissues and alveolar bone surrounding the teeth [1]. If left untreated, the inflammatory process may lead to bone loss, and eventually tooth loss [1]. Furthermore, periodontitis has shown associations with systemic diseases such as diabetes and cardiovascular diseases and may increase the risk of complications of these diseases [2, 3]. Therefore, it is conceivable that periodontitis can have a significant impact on individuals' general health and quality of life. The prevalence of periodontitis has been reported to increase with age [4–7]. In addition to an increase in the proportion of older individuals in western industrialized countries [8], more people are retaining their natural teeth [9–11]. Therefore, the burden of periodontitis is expected to increase in the years to come.

In Norway, patients' expenses for periodontal treatment have partly been subsidized by the public health insurance system for two decades [12]. In addition, the number of patients per dentist in Norway is lower compared to most countries in the world and a large proportion of Norwegian dental practitioners have reported that they have an insufficient number of patients [13]. Despite this public financial support and readily accessible dental health services, a recent study on the same sample population as the present showed a periodontitis prevalence of 52.6% [14]. This is within the range of other recent studies from Norway (33–81%) [4, 5, 15, 16]. The wide range in prevalence data from Norway may be due to different age groups included in the studies, and differences in living conditions with respect to availability of dental healthcare services, but also health behavior and socioeconomic status throughout the country.

In previous studies, smoking has shown to be an important risk factor for periodontitis [4, 6, 15–17]. In addition, male gender [7, 16], lower levels of education [4, 7] and self-reported diabetes [6] have been associated with the disease. A study from U.S. also showed ethnic differences in the risk for periodontitis, with increased risk among Mexican Americans and non-Hispanic black people [6]. Data regarding the association between periodontitis and dental attendance pattern are inconsistent [4, 6, 16]. In addition, previous studies from Norway have also found periodontitis to be associated with lower levels of education [4, 16], and rural living area [4]. These studies were performed in the northern part of Norway, and the self-assessed dental health and lifestyle habits have been reported to be poorer in these parts compared to the population of Oslo, the capital of Norway [18]. Furthermore, a higher prevalence of periodontitis among comparable age groups have been reported in these studies [4, 16] compared to the present sample population [14]. It is therefore conceivable that differences in living conditions can lead to differences in significance of risk factors for the development of periodontitis. However, data regarding socioeconomic and behavioral factors related to periodontitis in urban, senior citizens with easily accessible dental services as the present study population, are limited. Therefore, data from the present study will be useful in predicting individuals at risk of developing periodontitis and thereby individuals in need of preventive measures.

In addition to investigating possible risk indicators for periodontitis, it is important to consider if the disease affects individuals´ quality of life (QoL). The World Health Organization has defined QoL as “individuals' perception of their position in life in the context of the culture and value systems in which they live and in relation to their goals, expectations, standards and concerns” [19], and this parameter has been recognized as a valid assessment in both physical and mental healthcare, including oral health [20]. Several instruments for measuring oral health-related quality of life (OHRQoL) have been used in previous studies [21]. The Oral Health Impact Profile-14 (OHIP-14) is one of the most commonly used, validated self-reported questionnaires measuring impact of oral diseases on individual’s quality of life [22, 23]. A systematic review investigating periodontitis’ effect on OHRQoL showed inconsistent results [24] indicating a need for further studies. Moreover, studies included in the review used different periodontal examination methods and the populations investigated were heterogeneous. Furthermore, additional studies investigating the association between OHRQoL and periodontitis severity according to the 2017 World Workshop on the Classification of Periodontal and Peri-implant Diseases and Conditions [25, 26] in general populations has been requested [27, 28].

Therefore, the aims of the present study were to investigate socioeconomic and behavioral risk indicators for severe periodontitis in a random sample of 65-year-olds in Oslo, and to investigate if periodontitis has an impact on the OHRQoL in this young elderly population.

## Materials and methods

### Participants

In this cross-sectional study, periodontal disease in a 65-year-old population in Norway was investigated. A random sample of 460 individuals, 65 years of age (born in 1954) and residing in Oslo was drawn from the Norwegian Population Register (retrieved from the Norwegian Tax Administration). Inclusion criteria were “born in 1954” and “resident in Oslo”. In order to detect and document oral conditions with a prevalence of at least 10%, and the possibility for longitudinal follow-up after 5 years, the final sample size estimate was 450 individuals. The recruitment procedure has been described in detail in previous publications [14, 29]. The study was approved by the Norwegian Regional Committee for Medical and Health Research Ethics (REK 2018/1383), and performed in compliance with the tenets of the Declaration of Helsinki. A written informed consent was signed by all participants prior to the clinical examination.

### Questionnaire

All participants answered a semi-structured questionnaire prior to the clinical examination using the Nettskjema software (University of Oslo, Norway). The questionnaire contained items regarding socioeconomic background, general diseases, medication use, smoking habits and regularity of dental visits. The following questionnaire items have been described in a previous publication [14]. Self-reported diabetes type 2 was assessed by yes/no questions. Smoking habits were assessed by the three response alternatives: “never smoker”, “former smoker”, “current smoker”. “Current smoker” was defined as an individual who smoked at least one cigarette daily. Current smokers also reported the number of cigarettes daily consumed. The participants’ country of birth was dichotomized into ‘western’ (Nordic countries, Western Europe, North America and Australia) and ‘non-western’ (the rest of the world). Level of education was dichotomized into ‘higher education’ (university/college education) and ‘lower education’ (high school, elementary school, or lower). Utilization of dental services was dichotomized into regular (at least every second years) and non-regular (occasional, pain or other urgent needs, never).

Oral health related quality of life was assessed using the shortened Norwegian version of the Oral Health Impact Profile (OHIP-14) [22, 23]. The questionnaire consists of 14 items divided into 7 dimensions. Ratings were made on a 5-point Likert scale for each item: 0 = never, 1 = hardly ever, 2 = occasionally, 3 = fairly often and 4 = very often, with sum score ranging from 0–56. Higher OHIP-14 scores indicate poorer OHRQoL. Participants reporting a negative impact (response codes: 3 'fairly often' and 4 'very often') on one or more of the 14 items were categorized as having negative impact on OHRQoL, while those who had response codes only from 0 to 2 in all items were considered as having fair OHRQoL by OHIP-14.

### Clinical and radiographic examinations

Two trained, calibrated dentists (ATTS and MTD) performed all clinical examinations at the Research Clinic at the Faculty of Dentistry, University of Oslo from February to December 2019. The clinical examination included registration of missing teeth, periodontal probing depth (PPD), bleeding on probing (BoP), furcation involvement and tooth mobility. The intra-class correlation coefficient (ICC) (95% CI) calculated using pocket depth registrations from seven participants (336 values per examiner) was 0.82 (0.78–0.86). Orthopantograms (OPG) and two horizontal bitewings (BW) were taken of all participants. The OPGs were obtained using a panoramic imaging unit (ProMax X-ray Dimax 3 and Planmeca ProOne, Planmeca Oy, Helsinki) at the Department of Maxillofacial Radiology at the Faculty of Dentistry, University of Oslo. BW were obtained using an intraoral imaging unit (MINIRAY, SOREDEX, PaloDEx Group Oy, Tuusula, Finland) with a rectangular collimator (length 30.5 cm). The periodontal status was assessed based on the consensus report from 2017 World Workshop on the Classification of Periodontal and Peri-implant Diseases and Conditions (2018 EFP/AAP classification) [25, 26], and the examination protocol and reproducibility of the examiners have been described in detail in a previous publication [14]. Briefly, for % radiographic bone loss the ICC (95% CI) was 0.79 (0.66–0.86) for inter-examiner agreement and 0.88 (0.86–0.90) for intra-examiner agreement, and for staging periodontitis the weighted Cohen’s kappa (95% CI) was 0.72 (0.66–0.78) for inter-examiner agreement and 0.90 (0.82–0.98) for inter-examiner agreement. To study the relationship between periodontitis (outcome variable) and socioeconomic and behavioral factors (independent variables), periodontal status was dichotomized into non/mild/moderate periodontitis (non-periodontitis participants, stage I or II periodontitis) and severe periodontitis (stage III or IV periodontitis).

### Statistical analyses

Clinical and radiographic registrations were collected in The Oral Data Collector sheet specifically designed for data entry in this study, developed in Microsoft Excel 2016 (Microsoft Corporation, Redmond, Washington, US), and imported into STATA (Stata version 16.1; College Station, TX, USA) for statistical analysis. Data were stored in Service for Sensitive Data (TSD facilities, UiO). Participants with < 2 remaining teeth were excluded from the analyses. The above-mentioned methods have been described in a previous publication [14]. The results from the descriptive analyses are presented as percentage distributions, mean and standard deviation (SD) or median and inter quartile range (IQR). Chi-square and Fisher's exact test were used to determine any differences in the distribution of categorical variables. As the continuous variables did not follow a normal distribution, Kruskal–Wallis ANOVA and the Mann–Whitney U-test were used to detect differences in median values between two or three groups. Univariate and multivariate logistic regression analysis were used to further explore the relationship between severe periodontitis (outcome variable) and socioeconomic and behavioral factors (independent variables). All exposure variables were included in the multivariate regression model. The results from the regression analyses are presented in the form of unadjusted and adjusted odds ratios (OR) with their 95% CI. The level of significance was set to *p* < 0.05.

## Results

### Risk indicators for severe periodontitis

Of 796 eligible participants, 460 individuals agreed to participate in the present study (response rate 58%). Seven participants were excluded from the analyses due to < 2 remaining teeth (n = 3) or missing questionnaire (n = 4), resulting in a study sample of 453 individuals. The recruitment process has been described in detail in a previous publication [14]. One of the participants that was excluded due to missing questionnaire had severe periodontitis, therefore the prevalence of periodontitis (52.5%) and severe periodontitis (36.0%) in the present paper differ slightly from the previous publication (52.6% and 36.1% respectively) [14]. The mean number of teeth in the present study was 25.6 (SD: 3.4). The prevalence of periodontitis was 52.6%, and severe periodontitis was found in 36.1% of the participants [14]. According to the clinical examination, 72.9% of the participants had at least one site with PPD of ≥ 4 mm, and bleeding on probing was present in ≥ 10% of sites in 24.9% of the participants [14]. Distribution with respect to participants’ background characteristics and periodontal status is shown in Table 1. According to bivariate analyses, stage III or IV periodontitis was significantly associated with smoking, diabetes type 2, country of birth, education and dental visits (Table 2). Being smoker, having diabetes type 2 and born in a non-western country significantly increased the odds for stage III or IV periodontitis when the selected variables were included in the model (adjusted ORs).

**Table 1:** Background characteristics of study participants according to periodontal status

	Total	Non-/stage II periodontitis	Stage III or IV periodontitis
	n (%)	n (%)	n (%)
Total	453 (100)	290 (64.0)	163 (36.0)
Gender			
Men	233 (51.4)	144 (61.8)	89 (38.2)
Women	220 (48.6)	146 (66.4)	74 (33.6)
Country of birth			
Western	412 (91.0)	274 (66.5)	138 (33.5)^a^
Non-western	41 (9.1)	16 (39.0)	25 (61.0)^a^
Education			
Higher education	303 (66.9)	205 (67.7)	98 (32.3)^a^
Lower education	150 (33.1)	85 (56.7)	65 (43.3)^a^
Diabetes II			
Yes	29 (6.4)	10 (34.5)	19 (65.5)^a^
No	424 (93.6)	280 (66.0)	144 (34.0)^a^
Smoking			
Never smoker	197 (43.5)	146 (74.1)	51 (25.9)^a^
Former smoker	210 (46.4)	128 (61.0)	82 (39.1)^ab^
Current smoker	46 (10.2)	16 (34.8)	30 (65.2)^ab^
Dental visits			
Regular	404 (89.2)	270 (66.8)	134 (33.2)^a^
Not-regular	49 (10.8)	20 (40.8)	29 (59.2)^a^
Tooth brushing			
2 times daily	384 (84.8)	247 (64.3)	137 (35.7)
< 2 times daily	69 (15.2)	43 (62.3)	26 (37.7)

Letters in superscript indicates statistically significant difference between groups of same letter within the same variable. (*p* < 0.05: Pearson`s chi-squared test). N = 453

**Table 2:** Logistic regression model for severe periodontitis (stage III or IV)

Independent variables	Unadjusted	Adjusted
	OR (95% CI)	OR (95% CI)
Gender		
Men	1	1
Women	0.8 (0.6–1.2)	1.0 (0.6–1.5)
Country of birth		
Non-western	1	1
Western	0.3 (0.2–0.6)^a^	0.4 (0.2–0.9)^a^
Education		
Lower	1	1
Higher	0.6 (0.4–0.9)^a^	0.8 (0.5–1.3)
Diabetes II		
No	1	1
Yes	3.7 (1.7–8.2)^a^	2.5 (1.0–6.1)^a^
Smoking		
Never smoker	1	1
Former smoker	1.8 (1.2–2.8)^a^	1.9 (1.2–2.9)^a^
Current smoker	5.4 (2.7–10.7)^a^	5.8 (2.8–11.9)^a^
Dental visits		
Non-regular	1	1
Regular	0.3 (0.2–0.6)^a^	0.5 (0.2–1.0)
Tooth brushing		
2 times daily	1	1
< 2 times daily	0.9 (0.5–1.6)	1.6 (0.9–3.0)

*CI* confidence interval. Outcome variable: stage III or IV periodontitis

Letters in superscript indicates statistically significant difference from the reference category (*p* < 0.05). N = 453

### OHIP-14 total and domain scores

**Fig. 1:**
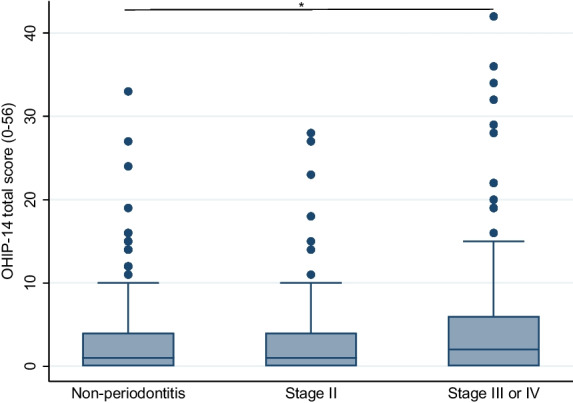
OHIP-14 total score in non-periodontitis participants, participants with stage II and stage III or IV periodontitis. Boxplots illustrating medians with interquartile ranges (IQRs) of OHIP-14 total scores in participants classified with non-periodontitis (n = 215), stage II periodontitis (n = 75) and stage III or IV periodontitis (n = 163). Kruskal–Wallis, Mann-Witney U test; *p < 0.05. Dots in the figure represent outliers. N = 453

Mean (SD) OHIP-14 total score in the sample population was 3.6 (6.1). With respect to periodontal status, the mean (SD) OHIP-14 total score was 2.9 (4.9) for non-periodontitis participants, 3.4 (6.0) among stage II periodontitis participants and 4.7 (7.4) among participants with stage III or IV periodontitis. Figure 1 shows the median OHIP-14 total score according to periodontal status. No participants in the present study sample were classified as having stage I periodontitis, this stage is therefore not included in the tables and figures below.

Mean and median OHIP-14 domain scores according to periodontal status are shown in Table 3. Psychological disability score and handicap score were significantly higher among participants with stage III or IV periodontitis compared to non-periodontitis participants. Physical pain score and psychological discomfort score were significantly higher among participants with stage III or IV periodontitis compared to non-periodontitis participants and participants with stage II periodontitis.

**Table 3:** Mean and median OHIP-14 domain scores according to periodontal status

	Non-periodontitis		Stage II		Stage III or IV	
	Mean (SD)	Median (IQR)	Mean (SD)	Median (IQR)	Mean (SD)	Median (IQR)
Functional limitation	0.4 (1.1)	0 (0)	0.5 (1.1)	0 (0)	0.6 (1.3)	0 (0)
Physical pain	0.9 (1.3)	0 (2)^a^	1.0 (1.5)	0 (2)^b^	1.3 (1.7)	1 (2)^ab^
Psychological discomfort	0.4 (0.9)	0 (0)^a^	0.3 (0.8)	0 (0)^b^	0.7 (1.5)	0 (1)^ab^
Physical disability	0.3 (0.8)	0 (0)	0.5 (1.1)	0 (1)	0.5 (1.2)	0 (1)
Psychological disability	0.4 (1.0)	0 (0)^a^	0.4 (1.0)	0 (0)	0.7 (1.4)	0 (1)^a^
Social disability	0.3 (0.8)	0 (0)	0.3 (0.8)	0 (0)	0.4 (1.1)	0 (0)
Handicap	0.2 (0.8)	0 (0)^a^	0.4 (1.1)	0 (0)	0.4 (1.1)	0 (0)^a^

Kruskal–Wallis ANOVA and Mann–Whitney U-test, sig if *p* < 0.05. Letters in superscript indicates statistically significant difference between groups of same letter on the same row. N = 453

### Frequency of negative impact on oral-health related quality of life

Negative impact on OHRQoL as defined in the present study (at least one question reported as fairly often/very often) was reported by 10.4% of the participants. The proportion of individuals reporting negative impact on OHRQoL according to the total OHIP-14 score did not significantly differ between the groups with different periodontal status (Table 4). When OHIP-14 domain scores were analysed separately, a significantly higher proportion of those with stage III or IV periodontitis compared to those without periodontitis or stage II periodontitis reported a negative impact on the OHRQoL in the psychological disability domain (Table 4).

**Table 4:** Percentage of participants who reported negative impact on OHRQoL (fairly often/very often on at least one domain question) according to periodontal status

	Non- periodontitis	Stage II	Stage III or IV
	n (%)	n (%)	n (%)
Total score	17 (7.9)	7 (9.3)	23 (14.1)
Functional limitation	9 (4.2)	4 (5.3)	11 (6.8)
Physical pain	7 (3.3)	4 (5.4)	10 (6.1)
Psychological discomfort	3 (1.0)	0 (0.0)	7 (4.3)
Physical disability	1 (0.5)	1 (1.3)	3 (1.8)
Psychological disability	2 (0.9)^a^	1 (1.3)	9 (5.5)^a^
Social disability	3 (1.4)	0 (0.0)	5 (3.1)
Handicap	4 (1.9)	1 (1.3)	5 (3.1)

Letters in superscript indicates statistically significant difference between groups of same letter within the same OHIP-14 domain. (*p* < 0.05: Pearson`s chi-squared/Fisher's exact test). N = 453

## Discussion

The present study was part of a larger epidemiological study investigating oral health among 65-year-olds in Oslo, Norway. While a previous publication has presented the prevalence of periodontitis in the present study population [12], this study aimed to investigate risk indicators for severe periodontitis and the disease’s impact on OHRQoL. The present study revealed several risk indicators for severe periodontitis in an urban Norwegian population of young elderly individuals. Despite the fact that the results indicated an overall good OHRQoL in this general population of young elderly, the OHRQoL decreased with increasing severity of periodontitis.

The results showed an increased risk for severe periodontitis among smokers, individuals with diabetes type 2, and individuals born in a non-western country. Current smokers had a five times increased odds ratio for severe periodontitis compared to never smokers. Smoking is a well-known risk factor for periodontitis, which has been described in previous literature [4, 6, 7, 16]. Because this risk indicator is modifiable, increased public knowledge and information to periodontitis patients may be of great significance in order to reduce the prevalence and progression of periodontitis in the population.

The increased risk for severe periodontitis among individuals with diabetes type 2 are also in line with previous studies [6, 27], confirming the importance of information regarding this association to diabetic patients and healthcare professionals who treat them. Ethnic differences in the risk for severe periodontal disease have previously been described [6]. The effect of country of birth on periodontitis risk found in the present study may have several explanations. Previous studies have identified several complicating factors when providing health care to migrants including language barriers, social deprivation and traumatic experiences, lack of familiarity with the health care system, cultural differences, different understandings of illness and treatment, negative attitudes among staff and patients, and lack of access to medical history [30, 31]. Paainen et al. reported that dentists experienced difficulties with communication with respect to information about periodontal diseases and importance of self-care in the treatment of periodontal diseases [31]. Furthermore, a previous study investigating dental attendance patterns among 65-year-olds in Norway and Sweden showed that irregular use of dental services was more common among individuals of foreign ethnicity [32]. In the present study, regular dental visits and higher education reduced the odds for severe periodontitis in the bivariate regression analyses. However, when including all the selected variables this significance disappeared while the increased risk for severe periodontitis among non-western participants, individuals with diabetes type 2 and smokers remained significant. This suggest that the effects of dental visits pattern and education were accounted for by variations in country of birth, diabetes type 2 and smoking habits variables.

Regarding OHRQoL, a decrease in the OHIP-14 total score with increasing severity of periodontal disease was observed, indicating that severe periodontitis had impact on OHRQoL. This is in line with previous studies [27, 28, 33, 34]. In addition, a significantly higher proportion of individuals with severe periodontitis reported negative impact in the psychological disability domain compared to the non-periodontitis group. Significantly higher scores were also reported by participants with severe periodontitis compared to non-periodontitis participants in the physical pain, psychological discomfort, psychological disability, and handicap domains. The literature is inconsistent regarding which domains that are associated with periodontitis [28, 33, 34], and different perception of poor oral health in different cultures might influence the domain scores [35]. In addition, studies investigating the dimensional structure of the OHIP-14 questionnaire have revealed differences in number of dimension factors in different study populations [36–39].

In recent studies investigating associations between periodontitis according to the 2018 EFP/AAP classification and OHRQoL, higher OHIP-14 scores and more negative impact on OHRQoL have been reported [28, 33, 34] compared to the present study. This may be due to different sample populations with respect to age, socioeconomic status, and cultural differences between countries. Previous studies investigating OHRQoL in general have reported lower OHRQoL among younger individuals compared to older individuals [23, 33]. This suggests that symptoms of oral diseases, like pain or tooth loss, are more detrimental to the quality of life in younger individuals [33], maybe due to differences in disease coping mechanisms. In the present study, only 65-year-olds were included which might partly explain the low OHIP-14 score compared to studies including younger age groups. In addition, several studies investigating associations between OHRQoL and periodontitis have been performed in samples of periodontitis patients [27, 28]. These participants may be more aware of their disease and complications related to the disease and may thereby report lower OHRQoL compared to samples from the general population like the present sample population. However, more studies investigating OHRQoL and severity of periodontitis according to the 2017 consensus report in general populations have been requested [27, 28]. One limitation when investigating associations between stages of periodontitis and OHRQoL in the general population, is the low prevalence of stage IV periodontitis. This is in contrast to a population of patients referred to a periodontitis clinic. Therefore, stage III and IV periodontitis was grouped and classified as severe periodontitis in the present study to increase the power in the analyses. Stage IV periodontitis include factors that might have a larger impact on the OHRQoL, for example more complex tooth loss, masticatory dysfunction and bite collapse [25, 27], compared to stage III periodontitis. Therefore, studies in the general population including a larger sample size, and thereby enough power to analysing stage IV periodontitis as a separate group, could have revealed a stronger association between periodontitis and OHRQoL.

Although the overall OHIP-14 total score and domain scores were low, higher scores among participants with severe periodontitis compared to those without periodontitis were found. This may indicate that a decrease in OHRQoL in the elderly population in Oslo will become an increasing challenge in the years to come with a higher proportion of elderly with retained, but periodontal impaired, natural teeth. In addition, the range in OHIP-14 total score was large both in non-periodontitis participants and in participants with periodontal disease, indicating that some participants experience a negative impact on OHRQoL despite the overall low mean score. Therefore, further analyses including other oral parameters should be performed in future studies.

The present study revealed several risk indicators for severe periodontitis which are important to highlight in order to ensure that information is conveyed to individuals at risk but also to medical and dental practitioners. In addition it is essential to address this issue to public authorities so that the availability and accessibility of dental care and treatment services are available to all groups of the society [40]. In Norway, individuals with periodontal disease was the largest patient group that received subsidized dental treatment in 2013 [41]. This may indicate that independent of socioeconomic status, periodontal treatment is easily accessible for this group of patients. Grytten et al. showed that higher education had a positive effect on the probability of receiving periodontal treatment and thereby subsidized dental care [41]. Therefore, it should be questioned if the financing model in its current form supports those who need it the most. Furthermore, it is crucial that information regarding the subsidy scheme is made more easily available to all groups of individuals in the society.

A limitation of the present study is the cross-sectional design. By using this design, the ability to assess causality between an exposure variable and outcome variable is lost. The optimal design in order to investigate causal relationships are longitudinal approaches. However, data from the present study can be used as a baseline for follow up studies. Furthermore, the OHIP-14 questionnaire used in the present study is a generic instrument rather than a disease-specific instrument [42, 43], and because this study was part of a larger epidemiological study, a generic instrument for measuring OHRQoL was used. It might be speculated that an instrument specifically designed to investigate the association between periodontitis and OHRQoL would be more sensitive to periodontitis specific problems. In addition, another limitation in the present study was the absence of validation of the dimensional structure of the OHIP-14 questionnaire in the present study population. Therefore, the association between periodontitis and certain domains of OHIP-14 in the present study should be interpreted with caution.

## Conclusions

The present study showed an increased odds for periodontitis stage III or IV in individuals born in a non-western country, individuals with diabetes type 2 and in smokers, indicating a need for information and attention to these groups in order to prevent increasing prevalence of severe periodontitis in the years to come. Overall, the results indicated a good OHRQoL among 65-year-olds in Oslo, however a tendency of reduced OHRQoL with increasing severity of periodontitis was observed.

## Data Availability

The datasets used and/or analyzed during the current study are available from the corresponding author upon reasonable request.
